# CircSLC8A1 and circNFIX can be used as auxiliary diagnostic markers for sudden cardiac death caused by acute ischemic heart disease

**DOI:** 10.1038/s41598-021-84056-5

**Published:** 2021-02-25

**Authors:** Meihui Tian, Jiajia Xue, Cuiyun Dai, Enzhu Jiang, Baoli Zhu, Hao Pang

**Affiliations:** 1 Department of Forensic Genetics and Biology, School of Forensic Medicine, China Medical University, No. 77 Puhe Road, Shenyang North New Area, Shenyang, 110122 P.R. China; 2 Department of Forensic Pathology, School of Forensic Medicine, China Medical University, No. 77 Puhe Road, Shenyang North New Area, Shenyang, 110122 P.R. China

**Keywords:** Biomarkers, Cardiology

## Abstract

Sudden cardiac death (SCD) caused by acute ischemic heart disease (IHD) is a major cause of sudden death worldwide. Circular RNAs (circRNAs) are abundant in the heart and play important roles in cardiovascular diseases, but the role of circRNAs as biomarkers in the forensic diagnosis of SCD caused by acute IHD remains poorly characterized. To investigate the potential of two heart-enriched circRNAs, circNFIX and circSLC8A1, we explored the expression of these two circRNAs in different kinds of commonly used IHD models, and further verified their expressions in forensic autopsy cases. The results from both the IHD rat and H9c2 cell models revealed that circSlc8a1 level was upregulated, while the circNfix level was elevated in the early stage of ischemia and subsequently downregulated. The time-dependent expression patterns of the two circRNAs suggested their potential as SCD biomarkers. In autopsy cases, the results showed that the expression of these two circRNAs in the myocardium with acute IHD-related SCDs corresponded to the observations in the ischemic models. Further analysis related to myocardial ischemia indicated that circSLC8A1 showed high sensitivity and specificity for myocardial infarction and was positively correlated with creatine kinase MB in pericardial fluid. Downregulated circNFIX level could indicate the ischemic myocardial damage, and it was negatively correlated with the coronary artery stenosis grade. The combination of circSLC8A1 and circNFIX had better performance to discriminate IHD-related SCDs. The results suggested that circSLC8A1 and circNFIX may be used as auxiliary diagnostic markers for SCD caused by acute IHD in forensic medicine.

## Introduction

Sudden cardiac death (SCD) refers to a sudden, unexpected death caused by the loss of consciousness, which occurs within one hour of symptom onset when witnessed, or if not witnessed, an unexpected death from a cardiovascular cause where the individual was observed to be alive within the previous 24 hours^1^. It is the leading cause of mortality and responsible for 50% of all deaths from cardiovascular disease^2^. The most common underlying abnormality associated with SCD is ischemic heart disease (IHD), while acute myocardial ischemia and acute myocardial infarction (AMI) are the primary causes^2–4^. In addition to coronary artery disease (CAD), other diseases that decrease coronary blood flow or increase myocardial oxygen consumption, such as coronary artery vasospasm and coronary artery anomalies, can also lead to myocardial ischemia or even the occurrence of myocardial infarction (MI), and eventually trigger sudden death^1^. In practice of forensic science, the diagnosis of acute myocardial ischemia and AMI mainly depends on the pathological morphology of the heart^5^. However, since death sometimes occurs in a matter of minutes or even seconds, some anatomical cases lack typical pathological changes, and identification must occur with the exclusion of other causes of death based on negative anatomy^6^. Therefore, the identification of functional indicators is important for the purposes of both in determining a clinical and forensic diagnosis of acute IHD caused SCD^3^.

To date, although various candidate biomarkers for SCD have been discovered, early diagnostic and therapeutic biomarkers remain scarce because of the complex pathophysiology and aetiology of SCD^7^. Some traditional blood markers, such as cardiac troponin I (cTnI), creatine kinase MB (CK-MB), and N-terminal pro-B-type natriuretic peptide (NT-proBNP), are commonly used in the clinical diagnosis of cardiovascular disease^8^. To avoid the influence of haemolysis, the levels of these functional biomarkers in the pericardial fluid are often used to assist the diagnosis of SCD postmortem, and immunohistochemical and genetic markers, which exhibit strengths and limitations, are also integrated into daily forensic practice^9–12^. With the development of RNA-sequencing technology and microarray analysis, growing evidence has revealed that circular RNAs (circRNAs) are abundant and widespread in the cardiovascular system, where they play a pivotal role in multiple developmental and pathophysiological processes of IHD^13–17^. CircRNA expression is tissue- and time-specific, and circRNA expression levels may be 10 times higher than those of linear RNAs^18^. In addition, circRNAs are significantly less susceptible to exonuclease activity than linear RNAs and can be secreted and identified in bodily fluids, which makes them promising candidate cardiac disease biomarkers^18,19^. For example, Vausort et al*.* found that the serum expression levels of myocardial infarction-associated circular RNA (MICRA) were lower in MI patients than in healthy volunteers and that the MICRA expression level might predict the extent of left ventricular dysfunction after MI^14,20^. Another study found that the expression level of cerebellar degeneration-related protein 1 antisense (CDR1as) in patients with AMI was positively correlated with the occurrence of ventricular arrhythmia^21^.

Studies have shown that two circRNAs, circSLC8A1 and circNFIX, are enriched in the myocardium in humans, mice (circslc8a1 and circnfix) and rats (circSlc8a1 and circNfix), and these circRNAs were shown to be differentially expressed in mice with IHD^22–24^. CircSlc8a1, which stems from the second exon of the arrhythmia-related sodium-calcium exchanger gene Slc8a1, is the most abundant circRNA in cardiomyocytes^16^. Previous studies showed that circSlc8a1 was upregulated in response to reactive oxygen species in the H9c2 myoblast cell line and promoted cardiomyocyte apoptosis by sponging miR-133a^25^. In addition, it was reported that reducing circSlc8a1 abundance attenuated ischemia-reperfusion (I/R)-induced cardiomyocyte apoptosis and MI, and circSlc8a1 may be a potential biomarker and risk factor predictor for myocardial I/R injury^23^. However, others reported that circSlc8a1 levels do not change during the myocardial stress response^24^. In contrast, circNfix was downregulated in the cardiac tissues of mice after infarction, and this downregulation in circNfix expression could promote cardiomyocyte proliferation and angiogenesis^22^. Cui et al. also found the expression of circNFIX was significantly downregulated in cardiomyocytes subjected to oxidative stress, and served as a pro-apoptosis factor in cardiomyocyte apoptosis^26^. According to the biological characteristics of circRNAs, we speculated these two differentially expressed circRNAs might have the potential to be biomarkers for forensic diagnosis of SCD caused by acute IHD. However, unlike samples under clinical application conditions, samples examined in forensic practice are often affected by postmortem changes^27,28^. Thus, the purpose of this study was to explore the potential of these two circRNAs as biomarkers for IHD-related SCD diagnosis in forensic autopsy cases. For this purpose, we first investigated the expression of these two circRNAs in different kinds of commonly used IHD models. Furthermore, we focused on verifying their expressions in forensic autopsy cases, and evaluated their application prospects in forensic medicine.

## Results

### Electrocardiogram tracings of acute IHD model rats

**Figure 1:**
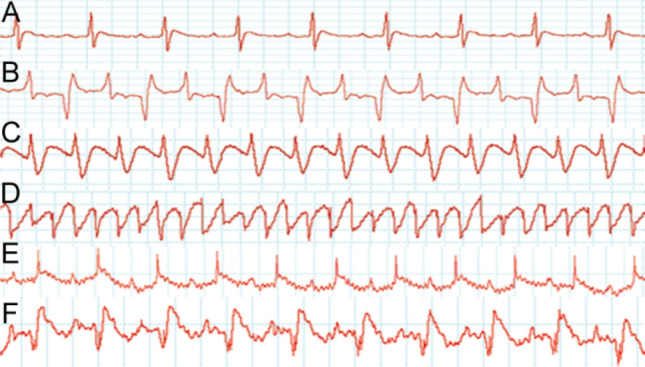
Electrocardiogram (ECG) tracings of rats in acute IHD models. The vertical axis of each ECG indicates voltage (mV), with 0.5 mV for all grids. The horizontal axis of each ECG indicates time (s), with 0.2 s for all four grids. **(A)** Normal rat ECG tracing. **(B–D)** ECG tracings of rats with ventricular arrhythmias (VAs) induced by BaCl_2_ solution: **(B)** supraventricular tachycardia (SVT), **(C)** ventricular tachycardia (VT), **and (D)** ventricular fibrillation (VF). **(E)** The ST segment elevation in rats after coronary artery ligation (CAL). **(F)** The ST segment elevation and pathological Q wave were shown in ECG tracings after continuous injection of isoproterenol (ISO) solution for 2 consecutive days.

The ECG tracings of rats from three acute IHD models are shown in Fig. 1. Normal electrocardiograms (ECGs) were recorded and shown in Fig. 1A. After the injection of BaCl_2_ solution, the ECGs of rats immediately showed the waveforms of ventricular arrhythmia (VA). Waveforms indicating ventricular tachycardia (VT) and supraventricular tachycardia (SVT) were the most common types of arrhythmias after injection of BaCl_2_ solution (Fig. 1B,C). All animals in the arrhythmia group died from ventricular fibrillation (VF) after a lethal dose of BaCl_2_ (Fig. 1D). The ECG tracings indicated that a model of VA induced by BaCl_2_ solution was successfully established in rats. In coronary artery ligation (CAL) rat models, after ligation of left coronary artery (LCA), the ST segment elevation was observed by ECG tracings, indicating the success of model construction (Fig. 1E). After injection of isoproterenol (ISO) solution for 2 consecutive days, the ECGs of rats showed changes in myocardial infarction (Fig. 1F).

### Histologic changes in the rat myocardium

**Figure 2:**
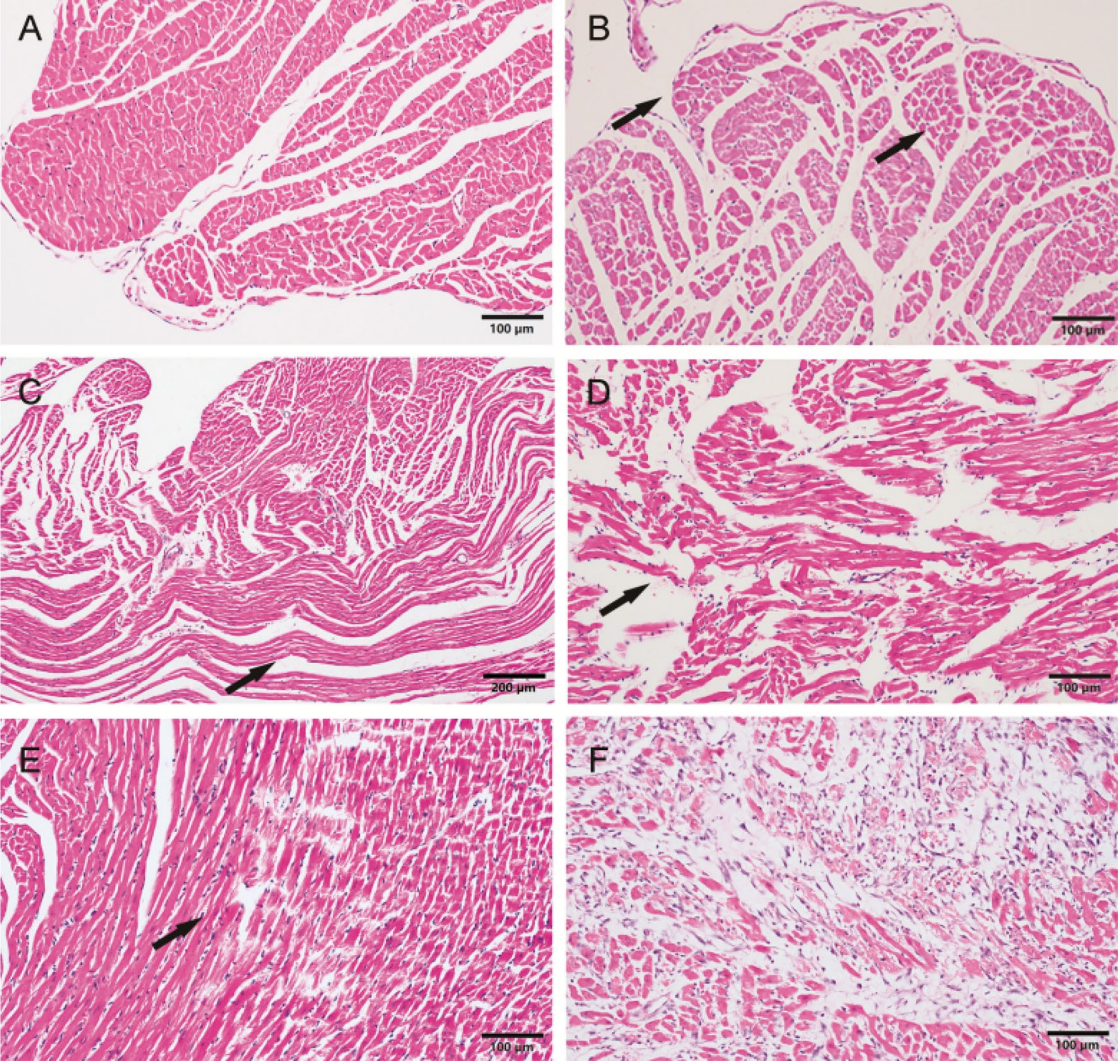
Haematoxylin–eosin (H-E) staining of the rat myocardium. (**A)** Normal rat left ventricular myocardium. (B) The left ventricular myocardium showed myocardial enhanced eosinophil staining (arrows) after 10 min of arrhythmia. **(C)** Enhanced eosinophil staining (arrow) was shown in the left myocardium after 10 min of arrhythmia in rat. **(D)** Myocardial wavy-like (arrow) was shown after 30 min of arrhythmia in rats. **(E)** Myocardial necrosis (arrow) with haemorrhage and contraction bans was shown after 60 min of CAL. **(F)** Coagulation necrosis with loss of nuclei and striations, interstitial infiltrate of neutrophils was shown after 2 consecutive-days injections of ISO solution.

We randomly selected three animals from each group in IHD models for histological examination, and the proportion of animals in IHD models that underwent histological examination was 84/170. The normal left ventricular myocardium of rats was shown in Fig. 2A. Enhanced eosinophil staining (Fig. 2B) and myocardial wavy-like (Fig. 2C) were the two most common nonspecific ischemic findings (proportion was 40/84 and 35/84, respectively) in rat myocardium Haematoxylin–eosin (H–E) staining of three acute IHD models. Myocardial interstitial haemorrhage (Fig. 2D) could be observed in the myocardium of rats with long period of arrhythmia and CAL (proportion: 9/84). After 3 h of coronary ligation, myocardial necrosis with hemorrhage and contraction bans (Fig. 2E) could be observed (proportion: 3/84). After injections of ISO solution for 2 consecutive days, gray-white infarcts were shown in the apex of rat hearts. Coagulation necrosis (Fig. 2F) with loss of nuclei and striations, interstitial infiltrate of neutrophils was shown in light microscopy (proportion: 3/14).

### Stability of two circRNAs after different postmortem intervals (PMIs)

**Figure 3:**
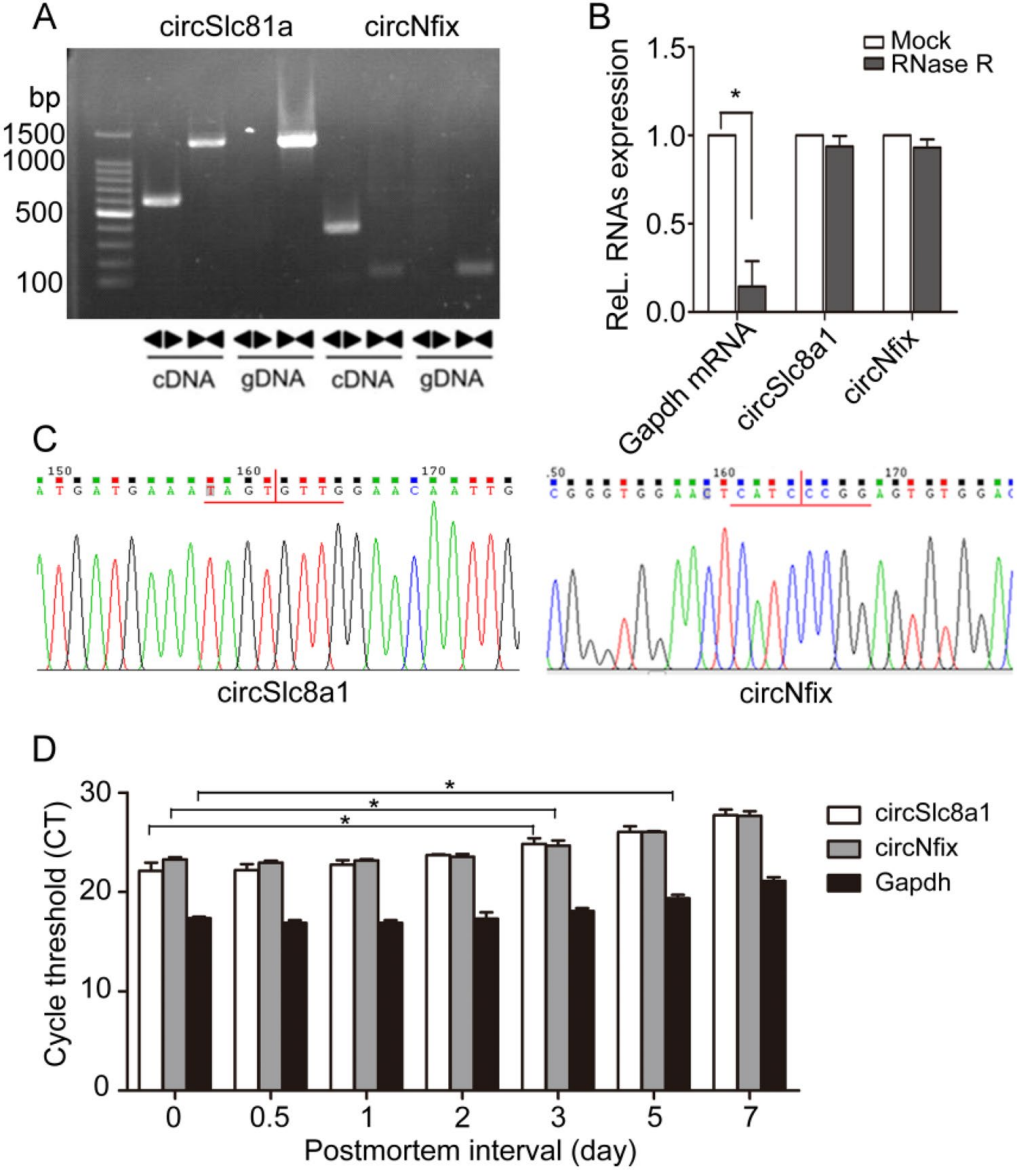
Verification of circSlc8a1 and circNfix. **(A)** CircSlc8a1 and circNfix are present in the myocardial tissues of rats. Clear single bands were amplified from the cDNA of rat myocardial tissue by divergent primers (◄►) and convergent primers (►◄), but could not be amplified by divergent primers from gDNA. Full-length gels are presented in Supplementary Fig. S1. online **(B)** Total RNA was incubated with RNase R or buffer only (Mock). After digestion, circSlc8a1, circNfix, and Gapdh mRNA was analysed by qRT-PCR. Differences between mock and RNase R were analyzed using student’s t-test, *P < 0.05, n = 3. **(C)** Sequence analysis validated the circular junction of the two circRNAs. **(D)** The CT values of circSlc8a1 and circNfix were determined by qRT-PCR at the corresponding timepoints. Differences between other timepoints group and 0-day group of each indicators were analyzed using one-way analysis of variance (ANOVA); post hoc analyses were performed using Dunnett’s multiple comparison test, *P < 0.05, n = 5.

Studies have reported that circSlc8a1 and circNfix are highly abundant and conserved in the cardiomyocytes of rats and humans^22,25^. Sanger sequencing of the amplified products of circSlc8a1 and circNfix by divergent primers and RNase R digestion assay were used to validate the circular nature of the circRNAs (Fig. 3A–C). To investigate the degradetion of these two circRNAs after different PMIs, we compared their expression in rat hearts by quantitative real-time polymerase chain reaction (RT-qPCR). The cycle threshold (CT) values of circSlc8a1 and circNfix in the myocardium were elevated on the third day after their placement in the artificial climatic chamber, while that of Gapdh mRNA was elevated on the 5th day (Fig. 3D). These results suggested that the target circular RNAs were less affected by postmortem changes within 3 days and could be used for subsequent research.

### Dynamic alterations of the two circRNAs of IHD models in vivo and in vitro

**Figure 4:**
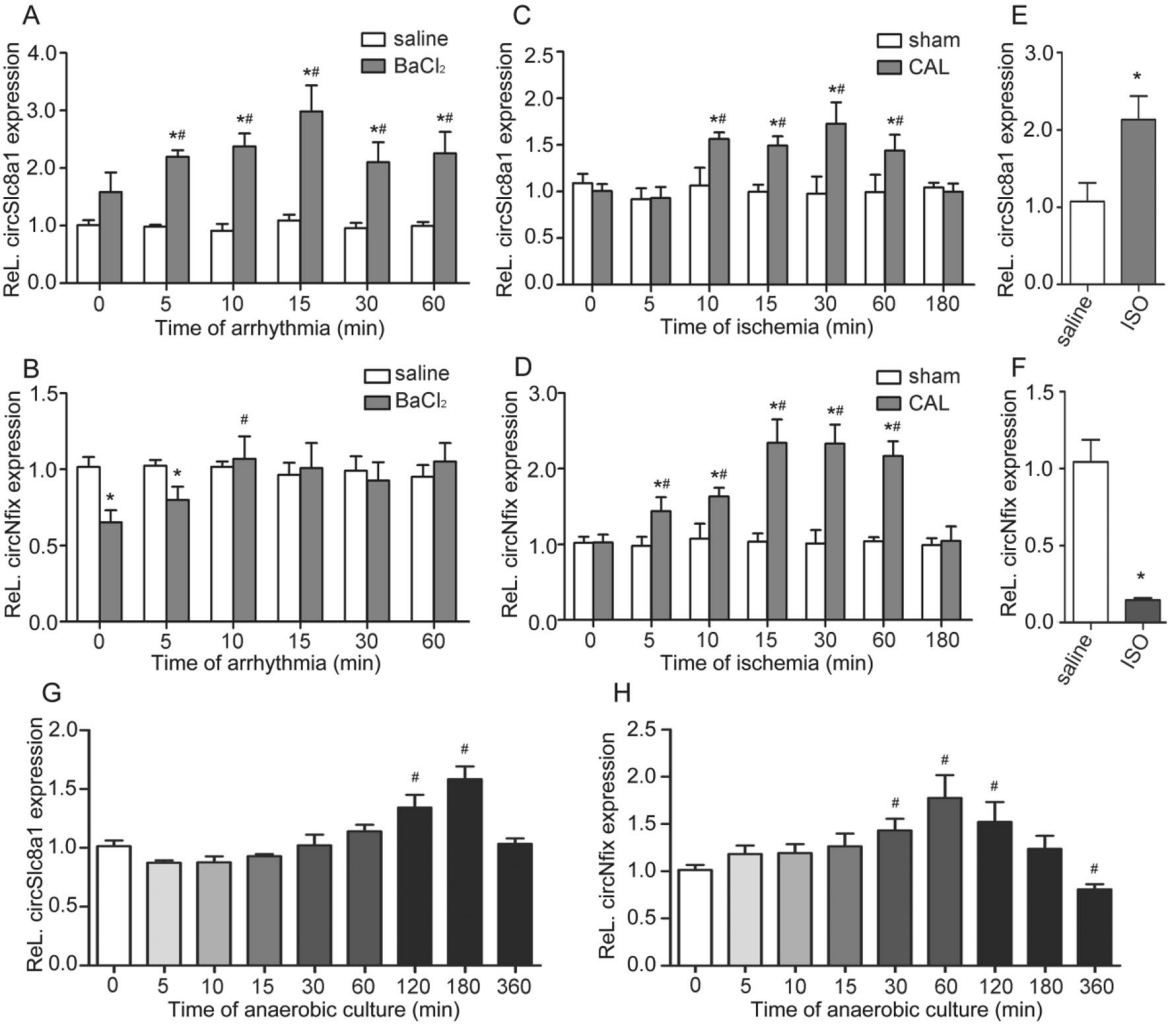
Expressions of circSlc8a1 and circNfix in the myocardial tissues of IHD rats. (**A, B)** CircSlc8a1 and circNfix expression in the myocardial tissues of rats after ventricular arrhythmia, n = 6. **(C, D)** CircSlc8a1 and circNfix expression in the myocardial tissues of rats after coronary artery ligation (CAL), n = 6. **(E, F)** CircSlc8a1 and circNfix expression in the myocardial tissues of rats at 48 h after the injection of ISO solution, n = 7. **(G, H)** Expressions of circSlc8a1 and circNfix in H9c2 cells treated with ischemia-hypoxia, n = 3. Differences between IHD treated group and saline group were analyzed using student’s t-test. Differences between other timepoints group and 0-min group of models were analyzed using one-way analysis of variance (ANOVA); post hoc analyses were performed using Dunnett’s multiple comparison test. *P < 0.05 (vs saline group), ^#^*P* < 0.05 (vs 0-min group).

The expressions of circSlc8a1 and circNfix in the cardiac tissues of rats from three IHD models were assessed by RT-qPCR. During VA in the arrhythmia model rats, circSlc8a1 levels in rats myocardium began to increase at 5 min and slightly decreased after reaching their peak at 15 min (Fig. 4A). The expression of circNfix in rats myocardium was significantly decreased compared with the saline group once arrhythmia occurred, and returned to normal level after arrhythmia for 10 min (Fig. 4B). In the myocardial ischemia model generated by CAL, the circSlc8a1 level in rats myocardium increased at 10 min after CAL, and this increase lasted for 60 min, after which the circSlc8a1 level significantly decreased at 180 min (Fig. 4C). circNfix levels in rats myocardium increased at 5 min after CAL, and changed in nearly the same manner as circSlc8a1 levels (Fig. 4D). In rats injected with ISO solution, the circSlc8a1 expression in rats myocardium was significantly elevated compared with that of the saline group, while changes in the level of circNfix were completely reversed (Fig. 4E,F).

Moreover, we detected the expression of the two circRNAs in H9c2 cells in anoxic environment by RT-qPCR. CircSlc8a1 levels increased after 120 min of ischemia-hypoxia treated in H9c2 cells, and decreased after peaking at 180 min (Fig. 4G). CircNfix levels increased after 30 min of ischemia-hypoxia treated in H9c2 cells and gradually decreased after reaching their peak at 60 min (Fig. 4H). After 360 min of treatment in anoxic environment, the circNfix expression in H9c2 cells was significantly downregulated compared with that in the 0 min group. The dynamic alterations of the two circRNAs of IHD models in vivo and in vitro indicated their potential as auxiliary indicator for the detection of SCD caused by IHD.

### CircRNA levels indicate the cause of death in forensic autopsy cases

**Figure 5:**
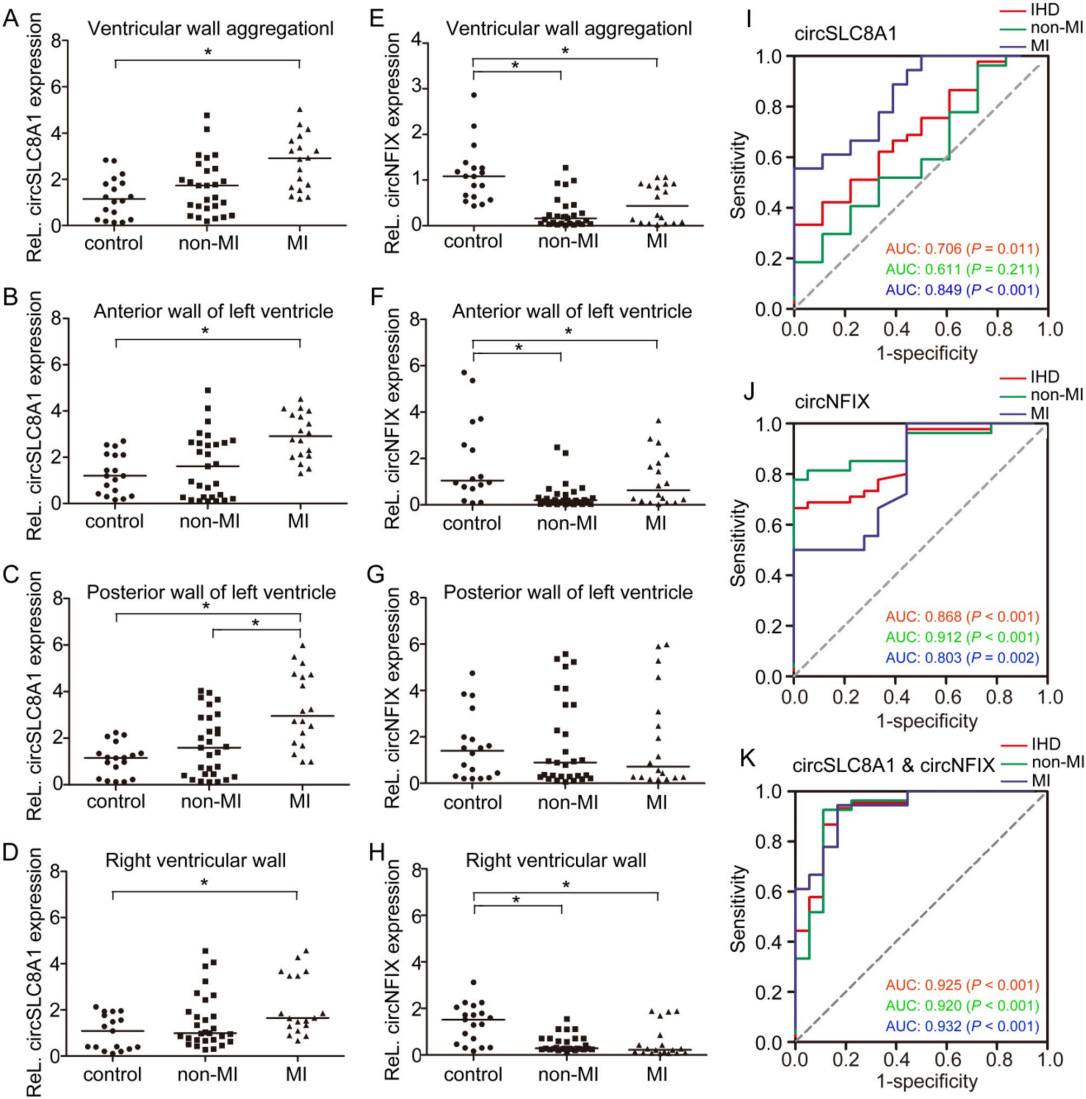
Relationship between circSLC8A1 and circNFIX quantification and the cause of death. Numbers in each group: control = 18; non-MI = 29; MI = 18. **(A–D)** Expression of circSLC8A1 in myocardium. The circRNA level in the whole ventricular wall was calculated based on the mean ΔCT value for each ventricular wall. **(E–H)** Expression of circNFIX in myocardium. **(I)** ROC curves of circSLC8A1. **(J)** ROC curves of circNFIX. **(K)** ROC curves of the combination of circSLC8A1 and circNFIX. Differences between groups were analyzed using Mann–Whitney U-test. * *P* < 0.05.

A total of 65 autopsy cases met the aforementioned inclusion criteria. According to the blood supply area of the left anterior descending coronary artery (LAD), left circumflex coronary artery (LCX), and right coronary artery (RCA) for the heart, we detected expressions of the circRNAs in myocardial tissues from the anterior wall of the left ventricle, posterior wall of the left ventricle, and right ventricular wall, respectively. SCD cases caused by acute IHD were divided into MI group and non-MI group, according to the cause of death based on forensic diagnosis. The correspondence between forensic autopsy cases and IHD rat models was shown in Supplementary Fig. S2 online. Compared to the control group, the MI group exhibited significantly higher circSLC8A1 expression, but the difference in circSLC8A1 expression between the non-MI group and control group was not significant (Fig. 5A–D). As for circNFIX, overall, its expression was significantly decreased in both the non-MI and MI groups compared with that in the control group, but there was no difference in circNFIX expression between the non-MI and MI groups (Fig. 5E). The circNFIX levels in cardiac tissues from the anterior wall of the left ventricle and right ventricle were consistent with the overall circNFIX level, but there was no significant difference in circNFIX level between these tissues and cardiac tissue from the posterior wall of the left ventricle (Fig. 5F–H).

The area under the receiver-operator characteristic (ROC) curve (AUC) of circSLC8A1 for all IHD cases was 0.706, the 95% confidence intervals (CI) was 0.571–0.842 (*P* = 0.011), and the sensitivity and specificity were 0.667 and 0.671, respectively (Fig. 5I). The AUC of circSLC8A1 in the MI group was 0.849 (95% CI: 0.727–0.971; *P* < 0.001), and the sensitivity and specificity for the MI group was 0.778 and 0.667, respectively. The AUC of circSLC8A1 in the non-MI group was 0.611 (95% CI: 0.442–0.780; *P* = 0. 211). The AUC of circNFIX for all IHD cases was 0.868 (95% CI: 0.779–0.956; *P* < 0.001), the sensitivity and specificity were 0.711 and 0.778, respectively (Fig. 5J). The AUC of circNFIX for non-MI and MI groups was 0.912 (95% CI: 0.828–0.995; *P* < 0. 001) and 0.803 (95% CI: 0.659–0.946; *P* = 0.002), respectively. In addition, we conducted ROC analysis for the combination of the two circRNAs to test the diagnostic value for IHD-related SCDs. As shown in Fig. 5K, the AUC was higher (AUC: 0.925, 95% CI: 0.848–0.998; *P* < 0. 001) when compared to each circRNA alone, the sensitivity and specificity were 0.933 and 0.833, respectively. The AUC of non-MI and MI groups was 0.920 (95% CI: 0.829–0.996; *P* < 0. 001) and 0.932 (95% CI: 0.855–0.999; *P* < 0.001), respectively. The results showed that the elevated expression of circSLC8A1 in myocardium might suggest formation of infarction, and the circNFIX levels were decreased with the occurrence of ischemic lesions. CircSLC8A1 and circNFIX could serve as potential biomarkers for IHD-related SCDs. The combination of these two circRNAs in the myocardial tissues might increase the value for SCD identification.

### CircRNA expressions are correlated with the coronary artery stenosis grades

**Figure 6:**
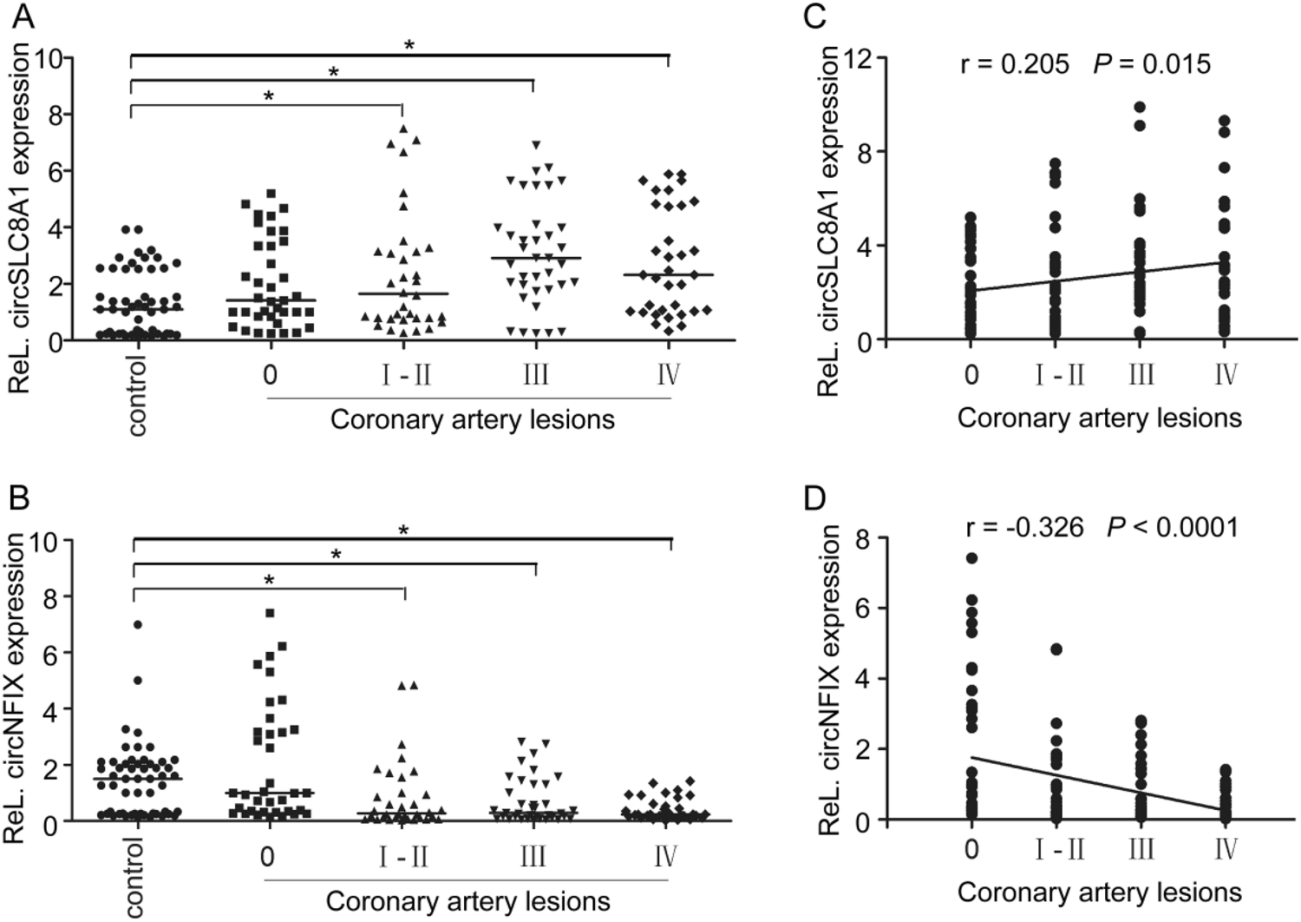
Correlation between circRNA levels and coronary stenosis grades. (**A, B)** Total circSLC8A1 and circNFIX levels in cardiac tissues supplied by three coronary arteries (numbers in each group: control = 54; 0 = 36; I—II = 34; III = 37; IV = 34). The circRNA level in cardiac tissues was calculated based on the mean ΔCT value for each coronary artery in controls. Differences between groups were analyzed using one-way analysis of variance (ANOVA); post hoc analyses were performed using Dunnett’s multiple comparison test, *P < 0.05. **(C, D)** Spearman plots of circSLC8A1 and circNFIX levels and coronary stenosis grades.

We hypothesized that the differences in circRNA expression among ventricular walls might relate to the degree of coronary artery stenosis (CAS). To verify this hypothesis, we regrouped the samples according to the stenosis grades of the three major coronary arteries (LAD, LCX, and RCA). The locations of coronary artery occlusion were measured within 2 cm from the beginning of the openings of each coronary artery, excluding the occlusion of the terminal branches of them. The CAS grades was determined according to the percentage of stenosis: grade I ≤ 25%, grade II 26–50%, grade III 51–75%, and grade IV ≥ 76%. Supplementary Fig. S3A–F online shows expressions of the two circRNAs in the myocardial tissues, which supplied by three coronary arteries with different grades of stenosis. The relative amounts of the two circRNAs in areas of the myocardium supplied by each coronary artery with different grades of stenosis were analysed collectively (Fig. 6A,B). Spearman correlation analysis was performed to assess whether the expression levels of the two circRNAs in the IHD group were related to the CAS grades. The results showed that the circSLC8A1 level was positively correlated with the grade of CAS to some extent (r = 0.205, *P* = 0.015, Fig. 6C), while the circNFIX level was negatively correlated with the grade of CAS (r = -0.326, *P* < 0.001, Fig. 6D).

### CircSLC8A1 levels in myocardium are correlated with the CK-MB levels in the pericardial fluid

**Figure 7:**
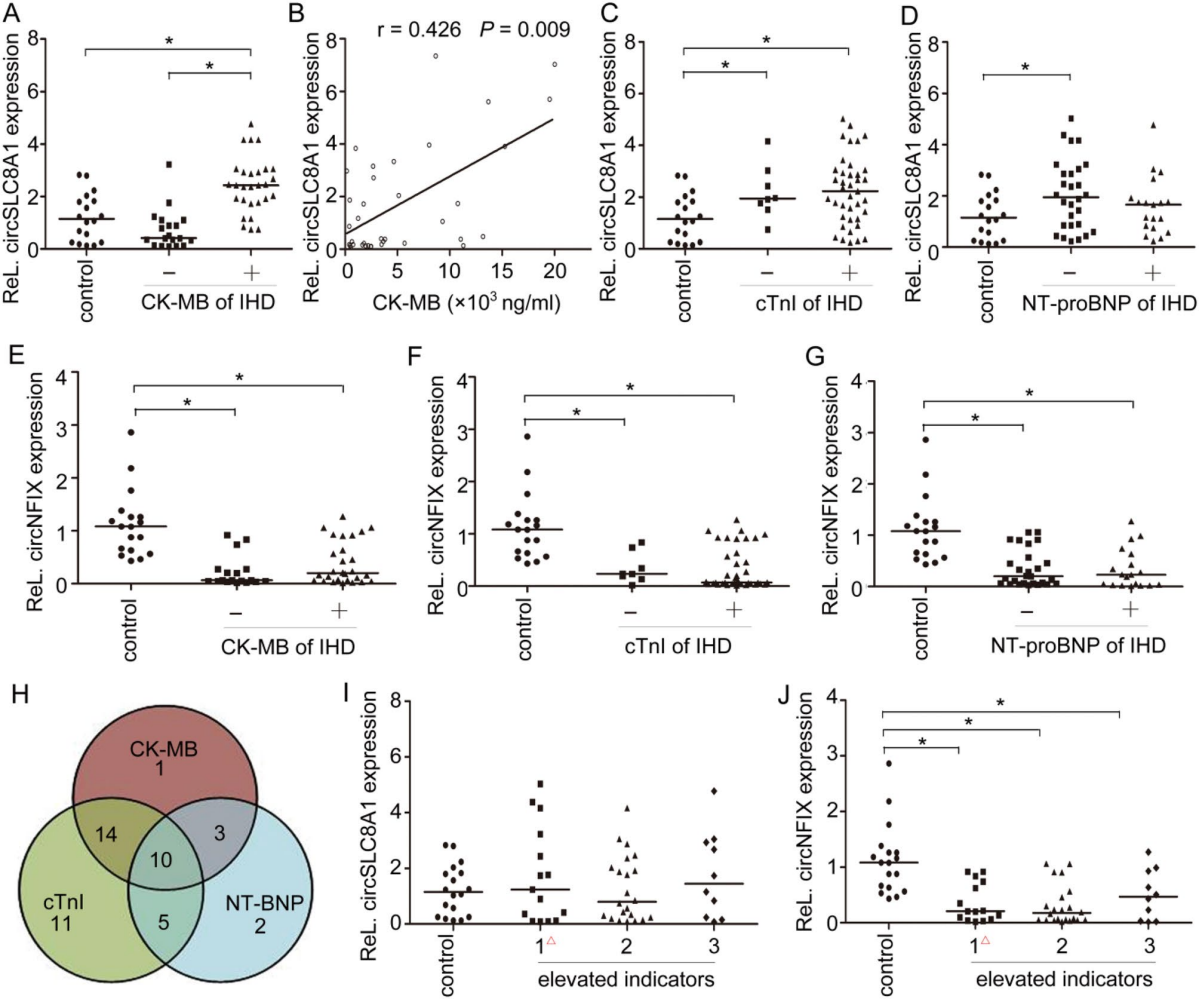
Relationships between the two circRNAs and traditional biomarkers in the pericardial fluid. CK-MB, creatine kinase MB; cTnI, cardiac troponin I; NT-proBNP, N-terminal pro-B-type natriuretic peptide. **(A)** CircSLC8A1 expression in cardiac tissues from decedents with different levels of CK-MB. Differences between groups were analyzed using Mann–Whitney U-test. * *P* < 0.05. **(B)** Scatter plot for the IHD group with CK-MB concentration on the X axis and circSLC8A1 expression on the Y axis. **(C)** CircSLC8A1 expression in cardiac tissues from decedents with different levels of cTnI and NT-proBNP in the pericardial fluid, Differences between groups were analyzed using Mann–Whitney U-test. * *P* < 0.05. **(E–G)** CircNFIX expression in cardiac tissues from decedents with different levels of CK-MB, cTnI and NT-proBNP in the pericardial fluid. Differences between groups were analyzed using Mann–Whitney U-test. * *P* < 0.05. **(H)** Venn diagram of biomarkers and their relationships. **(I, J)** Expression of circSLC8A1 and circNFIX in cardiac tissues from decedents with elevated levels of the indicated number of biomarkers (CK-MB, cTnI and NT-proBNP) in the pericardial fluid. ^△^ 0 or 1 biomarker whose levels were elevated. Differences between groups were analyzed using one-way analysis of variance (ANOVA); post hoc analyses were performed using Dunnett’s multiple comparison test, *P < 0.05.

Cardiac biomarkers, including cTnI, CK-MB, and NT-proBNP, are routinely used for the diagnosis of heart function. To investigate the relationships of the two circRNAs with these traditional biomarkers, the IHD cases were regrouped based on the traditional biomarkers levels in the pericardial fluid. The expression of the two circRNAs in the whole ventricular wall was analysed by RT-qPCR. In the IHD cases, decedents with elevated CK-MB levels in the pericardial fluid had higher expression of circSLC8A1 in the myocardial tissues than those with normal CK-MB levels (Fig. 7A). The circSLC8A1 level was moderately correlated with the CK-MB level in the pericardial fluid, the Spearman r was 0.426 (*P* = 0.009, Fig. 7B). Neither cTnI nor NT-proBNP levels in the IHD group showed a correlation with circSLC8A1 expression (Fig. 7C,D). Decedents with normal NT-proBNP levels in IHD cases showed significantly higher circSLC8A1 expression in the myocardium than those of the control group. Regardless of the levels of CK-MB, cTnI and NT-proBNP, the expression of circNFIX in the IHD group was lower than that in the control group, but there were no significant differences in circNFIX expression within the groups (Fig. 7E–G). Figure 7H shows the number of cases in the IHD group with abnormal levels of each indicator in the pericardial fluid. We also found that expression of the two circRNAs in cardiac tissues was independent of the number of elevated biomarkers (CK-MB, cTnI and NT-proBNP) in the pericardial fluid (Fig. 7I,J).

## Discussion

As lethal arrhythmia and AMI are often the first presentation of SCD in its victims, the detection of functional indicators for the clinical and postmortem diagnosis of SCD is an important, long-standing goal^29,30^. Although various candidate biomarkers for SCD have been discovered, early diagnostic and therapeutic biomarkers remain scarce due to the complex pathophysiology and aetiology of SCD^7^. In the present study, in order to investigate the potential of two heart-enriched circRNAs, circNFIX and circSLC8A1, as biomarkers in acute IHD-related SCDs, we explored the expression patterns of these two circRNAs in myocardium of rats with ischemic damages and H9c2 cells treated with ischemia-hypoxia. Furthermore, the postmortem diagnostic values of these two circRNAs were also tested in forensic autopsy cases. The circSLC8A1 expression was upregulated with ischemic damages, while circNFIX was downregulated. The expression of circSLC8A1 was positively correlated with the concentration of CK-MB in pericardial fluid, and circNFIX level was negatively correlated with CAS grade. Our data showed circSLC8A1 and circNFIX could be used as an auxiliary biomarker for diagnosing IHD-related SCDs in forensic medicine, and the combination of circSLC8A1 and circNFIX had the better performance to discriminate SCDs.

The ultimate mechanism of SCD is most often lethal arrhythmias, particularly VA (such as VF). In most cases, fatal arrhythmia is usually triggered by MI, however, it can also occur in the absence of structural cardiac pathology^31,32^. Generally, SCD in this condition was categorized in acute myocardial ischemia in forensic practice. Therefore, functional indicators that can prove the occurrence of lethal arrhythmias before SCD is important forensic diagnosis. Previous studies have reported some indicators, such as BNP and endothelin-1, were upregulated after 10 min of arrhythmia in myocardium of rats without structural myocardial damage^4,33^. The earliest expressions of other early markers, such as connexin 43 and JunB, were observed in the interval 15–30 min after CAL in rats’ myocardium^34^. The present study showed an ultra-early (5 min) alterations of the two circRNAs in myocardial tissues of rats with ischemic damage, which implied their potential as biomarker in acute IHD. Besides, there is substantial evidence for the fatal interaction of myocardial ischemia and VA in the occurrence of SCD. However, it is difficult to distinguish myocardial ischemia and VA due to their concurrence in most cases^32,35^. In addition, myocardial ischemia and arrhythmia have not unambiguously been diagnosed by the commonly used biomarkers on protein levels^3,4,8–10^. Thus, the circNfix level may provide a new target for analysing cause of death, either arrhythmia or ischemia. Furthermore, a previous study demonstrated that downregulation of circNfix could promote functional recovery after MI^22^. Thus, we speculate that the downregulation of circNfix in rats with early arrhythmia might have caused cardiac self-regulation due to stress.

One study has shown that circSlc8a1 was upregulated during myocardial ischemia in mice^25^, which was in accordance with our results. Nevertheless, the elevated expression of circSLC8A1 was only confirmed in the postmortem hearts with MI, rather than that without MI. In addition, the results suggested that circSLC8A1 exhibit high sensitivity and specificity for the diagnosis of MI postmortem and the association between circSLC8A1 and CK-MB levels was validated. Although circSLC8A1 levels were not correlated with cTnI or NT-proBNP levels in the SCD group, only those in the SCD group with deceased but normal NT-proBNP levels showed significantly higher expression of circSLC8A1 in the myocardium compared to that of the control group. Our previous studies^4^ demonstrated that cardiac dysfunction occurred after 30 min of rat arrhythmia induced by a BaCl_2_ solution, and other studies^36,37^ have found that with extended time of CAL, the animal's heart function decreased. In the present study, the level of circSlc8a1 in the myocardium of rats was slightly decreased after prolonged treatment, which was consistent with the findings in practical cases. Thus, we speculate that circSLC8A1 is also involved in the progression of cardiac dysfunction, but additional experiments are required to confirm this hypothesis.

Additionally, the postmortem diagnostic value of circNFIX in SCD induced by IHD has also been verified both in MI and non-MI cases. Interestingly enough, we also found circNFIX level in the myocardium was moderately negatively correlated with the degree of coronary artery lesions. Research has shown that the majority of SCD caused by AMI occur with stenosis of at least a moderate (> 50%) diameter in one of three main coronary arteries^38,39^. Currently, in forensic practice, the coronary artery lesions grades can be intuitively diagnosed by autopsy examination, while imaging test is more widely used in clinical practice^40^. Since some imaging examinations are invasive and relatively expensive, scholars have found novel serum biomarkers that also show a certain degree of correlation with the severity of coronary artery lesions, such as NT-proBNP, pentraxin 3 and the lipid ratio^41–43^. In addition, some microribonucleic acids (miRNAs) were significantly associated with the presence and severity of coronary artery lesions in patients with CAD^44–46^. These results are noteworthy, since an ideal biomarker should not only indicate the cause of death but also reflect the pathological processes that occurred, making it useful for diagnosing and predicting the occurrence of SCD in practical clinical and forensic applications^3,47^.

At present, forensic studies on circRNAs have mainly focused on estimation of the PMI and identification of body fluids^27,48^. Postmortem changes, particularly haemolysis and degradation, were the most immense challenges that limited the application of many clinical biomarkers in forensic medicine^49–51^. As for circRNAs, though the average half-life of circRNA can reach 50 h, many factors, such as the length of the target fragment, specificity of the primers and basal expression level have to be taken in account^49,52,53^. A previous study evaluated the stability of several circRNAs in mice within 8 days after death. Their results indicated that circ-AFF1 showed good stability in dead bodies, and could be used as reference gene^48,53^. However, real-world conditions are much more complicated than laboratory conditions, especially in human cases. Our results following detection of the stability of circSLC8A1 and circNFIX suggested that samples collected within 48 h after death could be effectively tested with the current primers. GAPDH mRNA showed better stability than these two circRNAs, which might be related to its higher basal expression level^53^. To the best of our knowledge, this study was the first to evaluate circRNAs as biomarkers of SCD caused by IHD in forensic diagnosis with human cases. Alterations in the expression of these two circRNAs provide additional information beyond conventional biomarkers in terms of the forensic diagnosis of IHD-related SCD.

This study has several limitations. First, although the IHD animal models were established according to standard methods, there are currently no suitable methods to accurately control the time of death in CAL model by SCD. Based on these, corresponding controls were set up to ensure the credibility of the data. Although a high numbers of animals were used in this study, which was also in accordance to 3R principle (reduction, replacement, and refinement). Second, due to the limited number of SCD victims with available PMI data, the number of samples in this present study was low. Although the altered expression of two circRNAs was shown in the myocardium of rats with arrhythmia and ultra-early myocardial ischemia, corresponding cases could not be found, which might affect further verification. Besides, because fatal arrhythmias are commonly deemed to be the mechanism of death rather than the cause of death in forensic medicine, thus we could not analyze them as a separate group, although we have reason to believe that some SCD cases with mild coronary artery lesion were included in non-MI group. Third, systemic ischemic diseases, such as mechanical asphyxia and haemorrhagic shock, should be excluded in subsequent studies. Increasing the sample size and number of other ischemia-related diseases examined will be considered in future studies to further investigate whether these two circRNAs can be applied in the diagnosis of SCD in forensic practice.

In conclusion, the different expression levels of circSLC8A1 and circNFIX distinguished between myocardial ischemia and arrhythmia and provided new ideas for the forensic diagnosis of SCD caused by IHD. The results indicated that new molecular markers may be complementary tools for the forensic diagnosis of early ischemic myocardial damage in cases of SCD, which might make it possible to determine the duration and severity of myocardial ischemia in SCD.

## Methods

### IHD animal experiments

Adult Sprague–Dawley (S-D) male rats weighing 300–350 g (purchased from the Animal Center of the China Medical University) were kept at controlled room temperature under a 6 a.m. to 6 p.m. light regime and fed regular pelleted rat chow and tap water ad libitum. Rats were placed in a supine position throughout experiments after being anaesthetized by intraperitoneal injection with 2% sodium pentobarbital (1.5 mg/100 g body weight). A total of 176 rats were sacrificed in the IHD experiments, including 6 failed animals which have been excluded (three for overdose anaesthesia, two for ligation failure, one for ventilator failure) .

#### Rat VA model

The rat VA model was established according to our previous study^4^. In brief, rats (n = 72) were randomly divided into BaCl_2_ and saline groups (each n = 36). Animals in BaCl_2_ group were intravenously injected with a 10% BaCl_2_ solution by a microinjector pump (ALC-IP900, Alcott Biotechnology Co. , Ltd., Shanghai, China) at a dose of 0.004 mL/100 g body weight after anaesthesia, and euthanized by a double dose of BaCl_2_ solution at the corresponding timepoints (0, 5, 10, 20, 30, 60 min, n = 6). Lethal arrhythmias at the 0 min group were induced by a lethal dose (0.008 mL/100 g) of BaCl_2_, whereas in other arrhythmia subgroups, the rats were injected with BaCl_2_ at every 10-min intervals until the end of the experiments by which a lethal dose was given. Saline group animals were injected with the same volume of saline and sacrificed by dislocation of the cervical vertebra.

#### Rat myocardial ischemia model

The myocardial ischemia model was established via ligation of the LAD^36^. Rats (n = 84) were divided into CAL and sham-operated groups (each n = 42). After the rat was anesthetized, we performed a left thoracotomy, and carefully identified and occluded the LAD. A successful LAD ligation is verified by visual inspection of the change in apex colour and the ST elevation in ECGs. The rats in sham-operated group only performed surgical operations without ligation. All the animals in this model were sacrificed by dislocation of the cervical vertebra at 0, 5, 10, 20, 30, 60 and 180 min (n = 6).

#### Rat AMI model

The rat AMI model^54^ was established via subcutaneous injection of ISO solution (85 mg/kg) with an interval of 24 h over two days (n = 7). Rats which were injected with the same volume of saline were set as controls (n = 7). Animals were sacrificed by dislocation of the cervical vertebra at 48 h after the first injection.

The ECG signals from lead II of each rat were recorded with a Powerlab biological signal processing system (AD Instrument, Bella Vista, Australia). The changes of ECGs in arrhythmia group and myocardial ischemia group were recorded during the whole course. For the ISO-induced AMI model, four ECG tracings were recorded: before ISO injection, after the first injection, after the second injection, and before death, each for 5 min.

All experiments performed in the present study strictly conformed to the *Guide for the Care and Use of Laboratory Animals* prepared by the Institute of Laboratory Animal Research and published by the National Institutes of Health (NIH Publication No. 86–23, Revised 1985). China Medical University ethics committee approved the study type in the experiments (approval No. 8167070800)**.** The animal treatment methods and protocols were also carried out according to the *Animals in Research: Reporting *In Vivo* Experiments* (ARRIVE) guidelines (http://www.nc3rs.org.uk/page.asp?id=1357) and *Guidelines for the Care and Use of Laboratory Animals*.

### Postmortem stability

To investigate the stability of the two circRNAs with different PMIs, forty-two healthy S-D male rats were sacrificed by cervical vertebra dislocation and placed in an artificial climate chamber at ambient temperature (25 °C). Myocardial tissues were collected after 0, 0.5, 1, 2, 3, 5, and 7 days (each n = 6).

### Cell culture and treatment

H9c2 cells (Cell Culture Center, Shanghai, China), a rat cardiomyoblast cell line, were cultured in high-glucose Dulbecco’s modified Eagle’s medium (DMEM; HyClone, USA) supplemented with 10% foetal bovine serum (FBS; Gibco, USA), penicillin (100 U/mL) and streptomycin (100 µg/mL) in a 5% CO_2_ incubator at 37 °C. Subsequently, the cells were cultured in an anoxia chamber (Heracell 150i, Thermo, USA) saturated with 92% N_2_, 5% CO_2_, and 3% O_2_ (v/v/v) at 37 °C for 0, 5, 10, 15, 30, 60, 120, 180, and 360 min in glucose-free DMEM (Gibco) without FBS^55^.

### Human tissue collection

Forensic autopsy cases of sudden death caused by traffic accident (control, n = 18) and SCD caused by IHD (n = 47) at our institute were examined. The survival time of all the decedents was less than 30 min, and the PMI was less than 48 h. The causes of death were determined by three forensic pathologists on the basis of a comprehensive medico-legal investigation, including autopsy examination, histological, toxicological and biochemical analyses. All cases with any poisoning, injuries or any other significant complications were excluded. All of the control victims were without any known cardiovascular disease according to anatomical and pathological findings, the results of postmortem biochemical detection and clinical records (if provided). SCD cases consisted of non-MI group (n = 29) and MI group (n = 18) according to whether there was a formation of myocardial infarction. The myocardial tissues of the anterior wall of the left ventricle, the posterior wall of the left ventricle and the right ventricle were collected depending on the blood supply area of three coronary arteries. Case profiles are summarized in Table 1.

**Table 1:** Case profiles.

Group	Number	Gender	Age (year)		Heart weight (g)		Combined lung weight (g)		CK-MB (ng/ml)^1^		cTnI (ng/ml)^2^		NT-proBNP (pg/ml)^3^		Coronary lesions
		M/F	Mean	Range	Mean	Range	Mean	Range	Mean	Range	Mean	Range	Mean	Range	(1/2/3)^4^
Control	18	16/2	46	26–68	334	260–377	1096	633–1621	1457	580–2086	1576	568–2680	209	200–254	0/0/0
**IHD**															
Non-MI	29	29/0	54	19–71	469	298–760	1478	1080–2330	6725	400–20,000	9034	700–12,000	1105	300–2818	14/9/6
MI	18	15/3	52	30–75	453	291–780	1349	804–1980	3771	160–20,000	8691	60–12,000	806	300–2768	10/4/4
Total	65	60/5	52	19–75	444	260–780	1379	633–2330	5121	160–20,000	8035	60–12,000	900	200–2818	–

^1^^–3^Reference value of each indicator: cTn I 181–3352 ng/ml; CK-MB 340–2420 ng/ml; NT-proBNP < 300 pg/ml.

^4^The number of cases with coronary stenosis grades (CAS) greater than II. For example, the coronary lesions (1/2/3) of non-MI group is 14/9/6, which means there are 14 cases that with one CAS greater than grade II, 9 cases with two CAS greater than grade II and 6 cases with three CAS greater than grade II.

All procedures performed in this study involving human participants were in accordance with the ethics committee of China Medical University (no. [2017]062), and with the 1964 Helsinki declaration and its later amendments or comparable ethical standards. All participants’ legal guardians gave their informed consent either orally and with a thumb print (if they could not write) or in writing after the study aims and procedures were carefully explained to them.

### H-E staining

Cardiac tissue specimens fixed in 4% paraformaldehyde were embedded in paraffin and dehydrated in an alcohol gradient and pellucidum in xylene. Each paraffin block was then cut into 5 µm thick sections and mounted onto slides with 3-aminopropyl-triethoxysilane. Sections were identified using H-E staining following the manufacturer’s instructions. Histological changes were observed at 200-fold magnification by light microscopy.

### Identification and validation of circRNAs

Total RNA was isolated from cardiocytes or cardiac tissues with AG RNAex Pro reagent (Accurate Biotechnology Co., Ltd., Hunan, China) according to the manufacturer’s instructions. Total RNA (10 μg) was incubated with 10 U Ribonuclease R (RNaseR; Epicentre, USA) at 37 °C for 10 min in 1 × buffer (provided with the enzyme). The head-to-tail junction of the two circRNAs was validated by polymerase chain reaction (PCR) with divergent primers, and convergent primers were used as a control. PCR product amplification was performed for 35 cycles, and the specificity of PCR amplification was confirmed by agarose gel electrophoresis and Sanger sequencing. The sequences of all primers used are listed in Supplementary Table 1.

### RT-qPCR

Purified RNA was reverse transcribed using an Evo M-MLV RT Kit with gDNA Clean for qPCR (Accurate Biotechnology Co., Ltd., Hunan, China) following the manufacturer’s instructions to generate a cDNA library. RT-qPCR was performed using a SYBR Green Premix Pro Taq HS qPCR kit (Accurate Biotechnology Co., Ltd., Hunan, China) on a Roche LightCycler 480II. Each amplification reaction contained 1 μg of cDNA sample, 5 μL of 2 × SYBR Green Pro Taq HS Premix and 0.4 μL of each primer (10 mM). RNase-free water was added to reach a 10 μL reaction volume. Initial denaturation was conducted at 95 °C for 30 s, followed by 40 cycles at 95 °C for 5 s, 60 °C for 30 s, and 72 °C for 30 s. Relative quantification was analysed using the 2 –ΔΔCT method to simultaneously test the two circRNAs and the endogenous reference (Gapdh mRNA). Reactions were run in triplicate with at least three biological replicates.

### Measurement of classical biomarker in pericardial fluid

Pericardial fluids were centrifuged at 3000 g for 15 min to collect the supernatant. The concentrations of NT-proBNP, CK-MB and cTnI in the pericardial fluid were measured in a fluorescence immunoquantitative analyzer (Getein1100, Getein Biotech, Inc., Nanjing, China) according to the manufacturer's instructions, respectively. The samples used to detect CK-MB and cTnI were diluted (× 200) with normal saline. The reference of each indicator was: NT-proBNP < 300 pg/ml; cTn I 181–3352 ng/ml; CK-MB 340–2420 ng/ml.

### Statistical analysis

The data are represented as means ± standard deviations (SDs) for continuous variables with normal distributions, as medians (interquartile range) for continuous variables with skewed distributions, and as frequencies (%) for categorical variables. Differences between two groups as normally distribute were assessed using two-tailed student’s t-test, and differences between two groups as non-normally distribute were assessed using Mann–Whitney U-test. Differences among groups, which were normally distributed variables, were analyzed by one-way analysis of variance (ANOVA). Post hoc analyses were performed using Dunnett’s multiple comparison tests. Evaluation of the value of circRNAs for the diagnosis of SCD caused by IHD was carried out with ROC curve analysis by AUC as the global discrimination value measure. Binary logistic regression analyses were conducted to evaluate the diagnostic value of the combination of two circRNAs. Correlations between the circRNA levels and other traditional targets were evaluated by Spearman’s rho correlation test. The results were presented as 95% CI. All statistical processing was performed using GraphPad Prism 6.0 software (GraphPad Software Inc., La Jolla, CA, USA). A *P* value < 0.05 indicated statistical significance.

## Supplementary information

Supplementary information.
