# Supplementary material for: CircSLC8A1 and circNFIX can be used as auxiliary diagnostic markers for sudden cardiac death caused by acute ischemic heart disease
Source: Sci Rep. 2021 Feb 25;11:4695. doi: 10.1038/s41598-021-84056-5 (PMC7907149; doi:10.1038/s41598-021-84056-5)
Supplement: Supplementary file 1 — Supplementary information. [file 41598_2021_84056_MOESM1_ESM.doc]

| **Supplementary Table 1. Primer sequences used for PCR** | | |
| --- | --- | --- |
| **Primer name** |  | **Sequence (5' - 3')** |
| rat-circSlc8a1 (convergent) | + | GACTGTGTCCAACCTGACCTTGATG |
|  | - | CACCTCCATGATGCCAATGCTCTC |
| rat-circSlc8a1 (PCR) | + | GAGAGCATTGGCATCATGGAGGTG |
|  | - | CATCAAGGTCAGGTTGGACACAGTC |
| rat-circSlc8a1 (RT-qPCR) | + | ATTGAAGGCACAGCCCGAGGTG |
|  | - | CCCATTGAAACATTGGGTGGGAGAC |
| rat-circNfix (convergent) | + | GCCGCTCCCCATCAGTA |
|  | - | TCCAACCCCGACCAGAA |
| rat-circNfix (PCR) | + | TCCAGCCACATCACATTG |
|  | - | TGAACCAGGTGTAGGAGAA |
| rat-circNfix (RT-qPCR) | + | CGGGATGCCCACTTCTGCTT |
|  | - | GACCTGGTCATGGTGATTTTGTTT |
| rat-Gapdh mRNA | + | GTCGTGGAGTCTACTGGCGT |
|  | - | TCGTGGTTCACACCCATCAC |
| human-circSLC8A1 | + | ATTGAAGGCACAGCCCGAGGTG |
|  | - | AAACCATCGAAGGGACTGCC |
| human-circNFIX | + | CGTCCAGCCACATCACATTG |
|  | - | TCCTTCGACATCCGCTTTTC |
| human-GAPDH mRNA | + | ACATCGCTCAGACACCATG |
|  | - | TGTAGTTGAGGTCAATGAAGGG |
|  |  | |


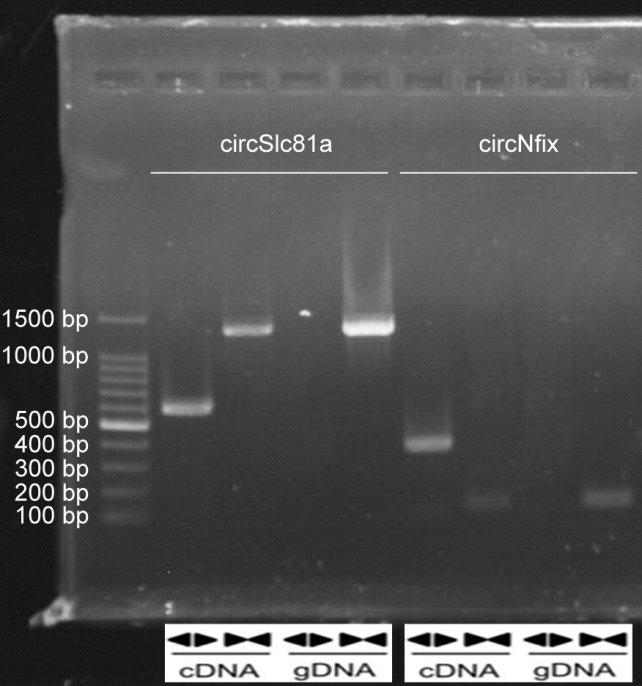


**Supplementary Figure 1. Full-length gels of Figure 3A in main text.** Divergent primers and convergent primers are represented by
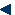

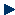
 and
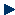

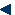
, respectively. CircSlc8a1 and circNfix are present in the myocardial tissues of rats. Clear single bands were amplified from the cDNA of rat myocardial tissue by divergent primers but could not be amplified from gDNA.

**
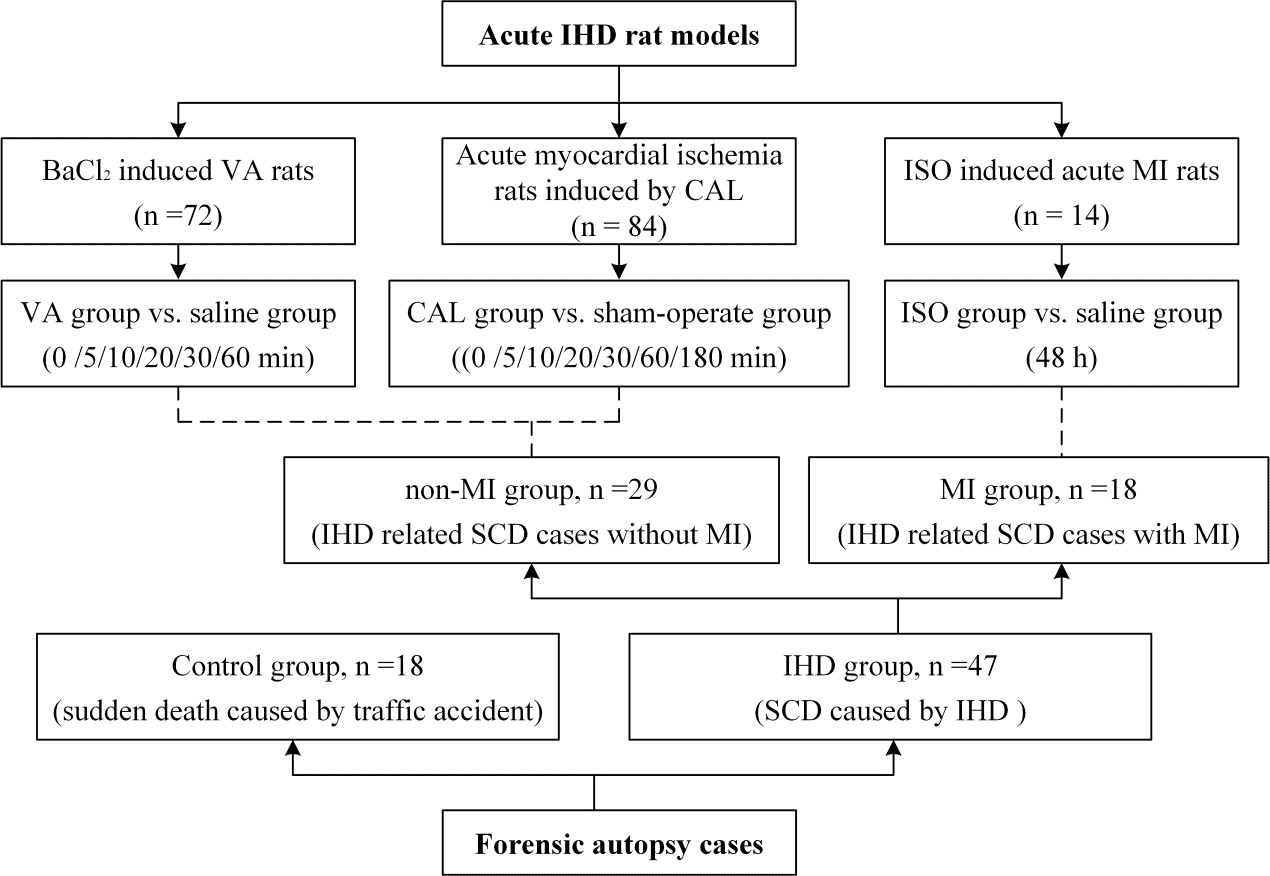
**

**Supplementary Figure 2. Graphical presentation of IHD rat models corresponding to autopsy SCD cases.** IHD: ischemic heart disease; VA: ventricular arrhythmia; ISO: isoproterenol; CAL: coronary artery ligation; MI: myocardial infarction; SCD: sudden cardiac death.


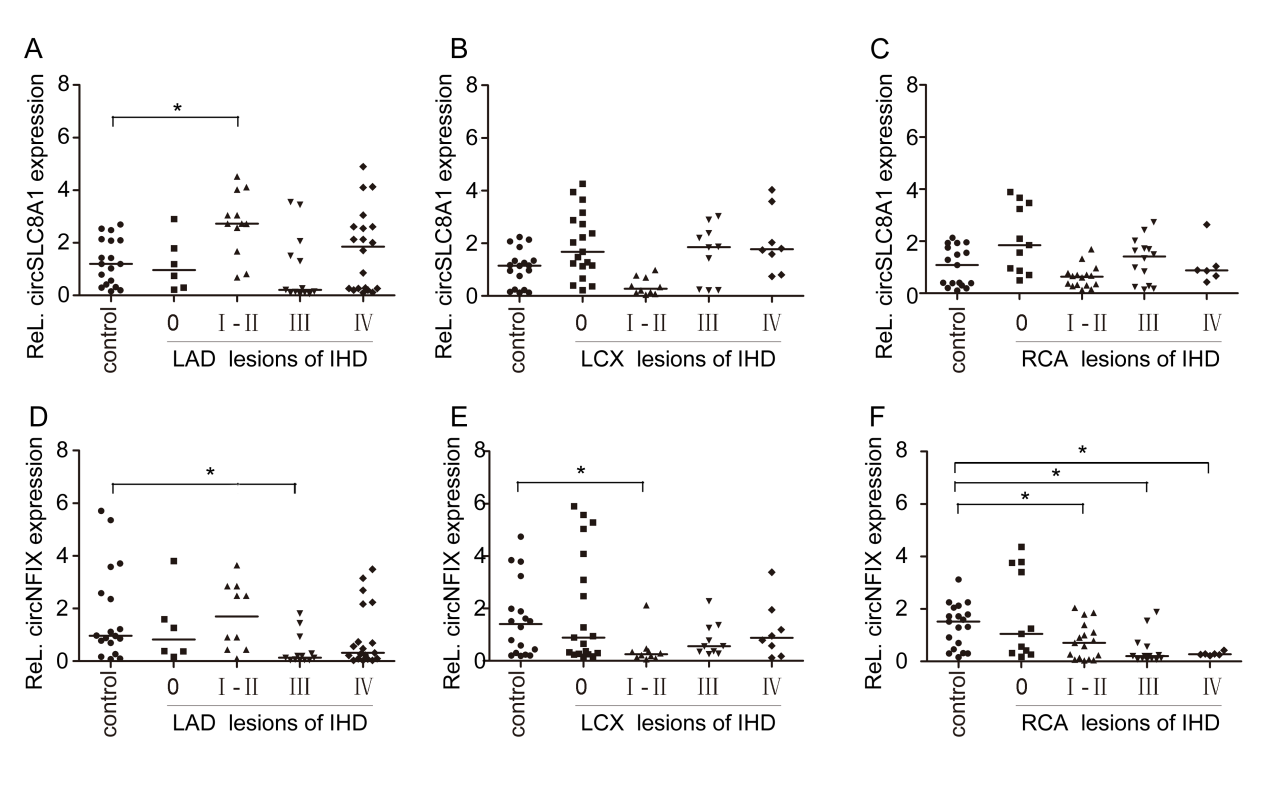


**Supplementary Figure 3. Expression of circRNAs in cardiac tissues with coronary artery stenosis.** LAD, left anterior descending coronary artery, numbers in each group: control = 18; 0 = 6; I - II = 8; III = 13; IV = 20; LCX, circumflex coronary artery, numbers in each group: control = 18; 0 = 19; I - II = 10; III = 10; IV = 8; RCA, right coronary artery, numbers in each group: control = 18; 0 = 11; I - II = 16; III = 14; IV = 6. **(A-C)** Expression of circSLC8A1 in cardiac tissues with coronary artery stenosis of the LAD, LCX and RCA. **(D-F)** Expression of circNFIX in cardiac tissues with coronary artery stenosis of the LAD, LCX and RCA. Differences between other timepoints group and 0-min group of models were analyzed using one-way analysis of variance (ANOVA); post hoc analyses were performed using Dunnett’s multiple comparison test, **P* < 0.05.
